# Following Gastrointestinal Surgery for Cancer: How Patients Pursue Surgical Treatment

**DOI:** 10.3390/bs16060842

**Published:** 2026-05-22

**Authors:** Eleonora Pinto, Gian Piero Turchi, Christian Moro, Alessandra Feltrin, Alessandro Fabbian, Genny Mattara, Pierluigi Pilati, Carlo Castoro, Rita Alfieri

**Affiliations:** 1 Hospital Psychology, Veneto Institute of Oncology IOV-IRCCS, 35128 Padua, Italy; alessandra.feltrin@iov.veneto.it; 2 Department of Philosophy, Sociology, Education and Applied Psychology, University of Padua, 35131 Padua, Italy; gianpiero.turchi@unipd.it (G.P.T.); christian.moro@unipd.it (C.M.); fabbian.alessandro@gmail.com (A.F.); 3 Unit of Surgical Oncology of the Esophagus and Digestive Tract, Veneto Institute of Oncology IOV-IRCCS, 35128 Padua, Italy; genny.mattara@iov.veneto.it (G.M.); pierluigi.pilati@iov.veneto.it (P.P.); 4 Division of Upper Gastrointestinal Surgery, Department of Surgery, Humanitas Research Hospital, 20089 Milan, Italy; carlo.castoro@humanitas.it (C.C.); alfieri.rita@humanitas.it (R.A.)

**Keywords:** discourse analysis, psycho-oncology, dialogic science, M.A.D.I.T. methodology, interaction, qualitative research, GI cancer, surgery, neoplasm, health

## Abstract

Previous studies have shown that, after postoperative recovery from upper and lower gastrointestinal surgery for cancer, patients use peculiar modalities to describe their health. The purpose of this study is to determine how upper and lower gastrointestinal cancer surgery is considered by patients when they set their health. A structured interview was developed and 47 consecutive patients were interviewed postoperatively. Answers were analyzed through M.A.D.I.T., a quantitative and qualitative methodology that allows for the detection of discursive processes comprising the text, beyond thematic analysis. Four dimensions have been analyzed: representation of the postoperative period in daily life; use of resources; participation in achieving the clinical objective after hospital discharge; and continuing to respect the surgeons’ indications. A corpus of 2374 text occurrences was analyzed. Without differences between types of surgery, surgical patients described the time after surgical intervention as a critical scenario. Patients expressed their personal opinions, expecting normality after surgery and having difficulty envisioning the future: their representation of inflexibility in the postoperative period prevented them from finding new coping strategies. Overall, across all four dimensions, participants used stabilization discursive modalities in more than 50% of cases, representative of a situation bound within strict ties and personal theories. When defining their health, cancer surgery patients tend not to consider their condition as a new and different one from before; they imagine that they will be able to fully resume their previous habits. However, this can risk undermining the achievement of the clinical objective. Thus, during early surgical consultations, as well as in surgical recovery, exploring differences after surgery and solutions could help patients in their engagement with surgical outcomes and consequences.

## 1. Introduction

The landscape of gastrointestinal oncology has undergone a radical transformation over the last decade. For patients diagnosed with esophageal, gastric, or colon cancer, the path to recovery is no longer a straight line. Instead, it has become a long journey of oncological therapies, biological challenges, and systemic physiological shifts. While these advancements have undeniably pushed the boundaries of survivability, they have also fundamentally altered the psychological burden placed upon the patient. Surgery is no longer just a physical event; it is the culmination of a demanding preparatory phase that can test a patient’s mental resilience, as much as their physical endurance. In the management of esophageal and gastric cancers, the clinical community has largely moved away from the “surgery-first” model. For patients affected by esophageal cancer, esophagectomy is a demanding and complex surgery that still presents high postoperative morbidity and significant long-lasting symptoms (Khalil et al., 2025).

Today, the standard of care for locally advanced cases involves intensive neoadjuvant regimens. By the time a patient arrives for an esophagectomy or a total gastrectomy, they have already endured months of nausea, nutritional depletion, and the chronic anxiety that accompanies every mid-treatment assessment. For these individuals, the transition from “cancer patient” to “surgical candidate” is accompanied by the fear that their body, already affected by toxicity, may not withstand the anatomical impact of a major resection (Benedict et al., 2016).

Concerning colorectal cancer and the related surgical treatment, even with the rise of robotic-assisted, minimally invasive colectomies that promise quicker physical recovery, the after-effects of the procedure remain significant (Morgan et al., 2023; Xi & Xu, 2021). The potential for a stoma—whether temporary or permanent—stands as one of the most significant psychological hurdle in GI surgery. It represents a threat to body image and social identity, often leading to preoperative depression that can, if unaddressed, translate into slower functional recovery and poorer adherence to postsurgical care.

Overall, data support multimodal treatment for upper and lower gastrointestinal cancers. As a result, an increasingly large cohort of these cancer patients live with surgical sequelae for many years. For them, improvement of long-term outcomes is still a challenge and, in the meantime, they face social, occupational and financial issues in the postoperative period, particularly during the first year after surgery (Khalil et al., 2025; McDermott et al., 2015).

In this regard, survivorship and related assets are becoming significant: not only do postoperative complications and comorbidities remain a major concern, but so do the ways in which patients respond to surgical stress and stimuli.

Interestingly, recent studies have shown that the health-related quality of life measurement—currently a cancer and oncological surgery outcome—is influenced by cancer adaptation and subjective judgements (Rupp et al., 2021). In this context, the long-term postoperative health-related quality of life reported by patients does not seem to be affected by the presence or absence of postoperative complications, nor is it reported differently according to differing levels of surgical complication severity (Markar et al., 2022; Konradsson et al., 2020). Rather, patients’ actions and intentions (for instance, physical activity, smoking, body weight management) aimed at mitigating the symptom’s burden after upper gastrointestinal surgery for cancer have been observed (Färnqvist et al., 2024).

Thus, field literature indicates that surgery invasiveness and the related adaptation of physiological functioning put patients at a high risk of postoperative complications. At the same time, patients’ health (Turchi et al., 2022b) and its consequent management contribute to improving or worsening their quality of life. In particular, since the postoperative period is life-challenging (Byrne et al., 2024; Turchi et al., 2022a), it is crucial to consider how patients who have undergone cancer surgery set their health, for instance through the modalities they use reporting on it.

In this respect, a rigorous definition of health applied to an oncological surgery setting, grounded on an adequate epistemological basis, is a configuration, generated within a dialogical process, that considers neoplasms and/or the implications of actions regarding neoplasms on an organic level as well as on an interactive level (Turchi et al., 2022b; Pinto et al., 2022). This means that considering health as a dialogic process entails the possibility of narrating health itself by considering several different aspects. Health is narrated in natural language and health narratives encompass physical conditions: health is not dichotomic (completely present versus completely absent), but different self-reported degrees (levels) of health are possible. Hence, in cancer patients subjected to surgery for upper and lower gastrointestinal neoplasms, a peculiar account of health with its own references and evaluation becomes possible.

As a result, the burden of postoperative disease and how the patient manages it play a part in pursuing the aim of the surgical treatment received (Turchi et al., 2022a, 2022b). Relying on data directly collected from interviews, the so-called qualitative research analyzes the experiences of patients comprehensively (Im et al., 2023), unveiling experiences and categories of knowledge used by patients in facing peculiar clinical conditions. Patients’ categories of knowledge of neoplasm and the care pathway can generally be taken for granted, but they become significant in adjustment to neoplasm and its treatments: for example, unmet needs that, when appreciated and answered, can enhance clinical intervention and a patient’s quality of life outcome. Exploring the modalities used by patients to form their health has been acknowledged as needful in the postoperative period (Gao et al., 2024). This requires us to bridge the gap concerning how patients configure a treatment with curative intent, rather than rating components of “quality of life” or clinical conditions. Therefore, the purpose of this study is to examine how upper and lower gastrointestinal surgery is considered by patients when they set their health.

## 2. Materials and Methods

According to the assumption that health is narrated, Dialogic Science was chosen as the elective approach for pursuing the study’s aim. Indeed, Dialogic Science deals with the sense-generation process that takes shape through natural language use, specifically through discursive modalities and contents (Pinto et al., 2022; Turchi et al., 2014). Analysis of discursive modalities and contents allows the exploration of configurations, in this case, for health configuration.

The Methodology for the Analysis of Computerized Text Data (M.A.D.I.T.)—a product of Dialogic Science—was used for text analysis, providing an examination of rhetorical-argumentative structures of narratives and an index for measuring the pragmatic impact of these narratives on people’s lives. M.A.D.I.T. is both a qualitative and quantitative methodology analyzing both the process of sense-generation (discursive modalities through which contents are argued) and contents (themes and specific dimensions of meaning). Specifically, discursive modalities are codified in 24 Discursive Repertories (DRs) (Turchi et al., 2021), whose definition is “a way of configuring reality, with pragmatic relevance and used as an establishment of truth, aimed at generating/maintaining a narrative coherence” (Turchi et al., 2022c).

DRs are organized in a Periodic and Semi-Radial Table and divided into three main categories: generative DRs, hybrid DRs and stabilization DRs. Their difference concerns the particular usage rules of natural language that account for different and peculiar narrative processes (of each single DR). In detail, generative DRs allow the possibility of varying the discursive process and the related realities of sense. Hybrid DRs endorse the capacity of the repertory with which they are associated, thus supporting the narrative process that is being created. Stabilization DRs establish a fixed reality of sense, hardly subject to change and tending to remain permanent.

Each DR has been assigned a value between 0.1 and 9.9, related to the generative, hybrid and stabilization categories. It is the dialogical weight (dW), which indicates a specific DR’s contribution in creating discursive configurations. Consequently, dW can be globally measured in texts answering a question, resulting in an index of the whole discursive configuration. In particular, low dW values (closer to 0.1) are measured in discursive configurations not subject to adjustment, while high dW values (closer to 9.9) are measured in discursive configurations with a high propensity for change. In addition to dW, the dialogical momentum (dM) is the theoretical ability of DRs to enter into relationships with one another, by virtue of the processual properties of each one of them: these relationships shape the argumentative structure and have repercussions on further interactions (e.g., adjusting for specific DRs). Both dW and dM are values measured on a numerical scale composed of specific values of dW and dM for each DR. In general, if the value is higher, the possibility of using various DRs leading to a complete discursive configuration of health will be greater, suggesting an adjustment to change in facing the oncological surgery.

For this purpose, consistently with the general aim of outlining how upper and lower gastrointestinal surgery is considered when patients set their health, specific objectives were defined to frame the questions (Table 1). In M.A.D.I.T., the question is a tool specifically designed for exploring how patients set their health. Moreover, the question allows the researcher to collect the category of construction of reality used by respondents and shown by the language modalities they use at that very moment about a specific timing (that can also be a different time, for instance, in the future). Indeed, the dialogical process of knowledge is placed within an incessant flow, and every time it manifests, it takes shape through natural language, its structure and contents.

Accordingly, a structured interview consisting of 4 open-ended questions was developed through 4 sub-objectives, with the aim of outlining how cancer patients represent the postoperative period in daily life; use their resources; participate in achieving the clinical objective after hospital discharge; and continue to respect the surgeons’ indications (Table 1).

Hence, in this cross-sectional study 47 consecutive patients were interviewed once. All patients had undergone esophageal–gastric or colorectal surgery for neoplasm, had been hospitalized from the 3rd postoperative day after surgery until hospital discharge, were able to answer, were proposed to participate in the study and were enrolled between April and June 2019. The third postoperative day was identified as the date from which patients were able to give their consent and answer questions: so, once out of the Intensive Care Unit, patients could be interviewed about their plans to face the postoperative period and navigate their return home. Indeed, for the postoperative period, patients were interviewed within the context of complications and precautionary measures, in order to examine their considerations about their own physical conditions and any precautionary measures suggested by health-carers, as well as their use of collected information, that may influence their return home and the postoperative period. Thus, a patient’s experience during the early postoperative period contains aspects that they use: they can reflect on and express them through language.

Informed consent was obtained from all study participants; the Institutional Ethical Committee approved all study materials and protocols. Answers were transcribed verbatim in a database and analyzed through M.A.D.I.T. methodology (Turchi et al., 2021), combining statistical and qualitative data to exploit peculiar forms of language (DRs), leading to different configurations. So, textual data were independently reviewed and coded line-by-line by two researchers (C.M. and E.P.) following the M.A.D.I.T. steps (Turchi et al., 2022c), after which coding discrepancies were discussed and any inconsistencies were resolved with a supervisor (G.P.T.) through the technique of triangulation aimed at controlling personal interpretation, values and prejudices. In the present study the analysis was performed manually using Excel software™. Coding consisted of two different groups of codes: one group concerning the processes (the different DRs), and the other group concerning the themes identified (contents). After this, the transcripts were re-evaluated and both the supervisor and researchers agreed on the final result, reducing the risk of possible biases in data coding, so as to provide the final DRs and coding content in a reliable and substantial way.

## 3. Results

We collected answers to four narrative questions from patients subjected to upper and lower gastrointestinal surgery.

Of the 47 patients, 17 were female and 30 were male; 45% of the total were younger than 66 years old and the remaining 55% were older (the mean age of respondents was 66.78 years, minimum value 43 and maximum value 90). Moreover, of these patients subjected to curative-intent cancer surgery, 62% underwent preoperative chemo or chemoradiotherapy (30% and 32%, respectively) while 38% underwent surgery directly as a first-line cancer treatment. Also, 18 patients had a diagnosis of esophageal cancer, 6 esophageal–gastric junction cancer, 14 gastric cancer, 4 colon cancer and 5 rectal cancer. Overall, 38 were therefore upper-GI cancer patients and 9 were lower-GI cancer patients. The surgical treatments carried out were esophagectomy (51%), gastrectomy or gastric resection (28%), colic resection or sigmoid colon resection (13%), anterior resection of rectum (6%) and the Miles procedure (2%).

Overall, a corpus of 2374 text occurrences was analyzed. Within the general aim (outlining how upper and lower gastrointestinal surgery is considered by patients when they set their health), the first question asked the patients to describe the postoperative impact on daily life.

### 3.1. Representing the Postoperative Period in Patient’s Daily Life

**Figure 1:**
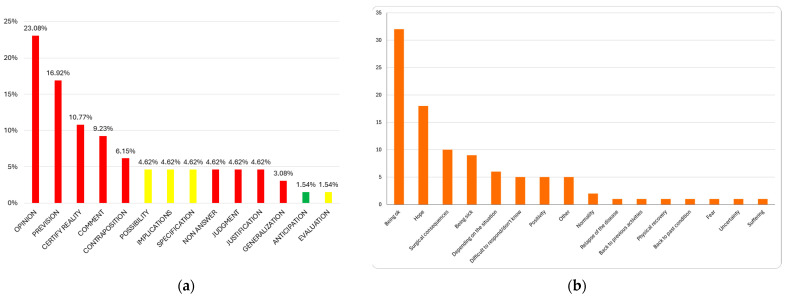
Question 1—Please imagine you are at the surgical outpatient clinic about 3 months after surgery: when the surgeon asks you to describe your physical condition with respect to surgery, what might your answer be? (**a**) Discursive repertories; (**b**) contents.

In this respect, when answering the question “Please imagine you are at the surgical outpatient clinic about 3 months after surgery: when the surgeon asks you to describe your physical condition with respect to surgery, what might your answer be?” a prevalence of stabilization repertories was observed. Indeed, as can be noted from Figure 1a, which shows the distribution of all DRs from the answer from the first question, stabilization DRs cover four-fifths of the total discursive modalities used by patients.

Specifically, the most frequent DRs were Opinion and Prevision. Hybrid repertories were used with a lower percentage (4.52% for Possibility, Implication, Specification and 1.54% for Evaluation), while generative repertories were used only once (Anticipation, 1.54%) (Figure 1).

In relation to themes and specific contents used (Figure 1b), “Being ok”, “hope”, “surgical consequences” and “being sick” were the ones mainly expressed in patients’ narratives regarding the postoperative configurations (Figure 1).

dW and dM were measured and had values of 1.6 and 4.7, respectively: these values highlight a global postoperative configuration characterized by personal opinions and forward-looking statements not subject to change (dW), as well as a low propensity to trigger other and different discursive modalities (dM). These results point out that the course of recovery is established by patients in the early postoperative period, with reduced uncertainty. Clinically, the modalities described in patients’ answers are aimed at reducing the burden of surgical treatment. When regarded as successful or, conversely, as ruled by neoplasm and its consequences, the representation of the postoperative period serves as a prototype for patients trying to deduce a series of actions or action–reaction patterns in order to adjust to the disease. Additionally, without describing and specifying problems and their reasons, these actions or patterns for adjustment are less effective.

### 3.2. Describing the Patient’s Use of Resources

The second question was aimed at capturing the patient’s use of resources to cope with clinical issues (“Consider the answer you gave to the previous question, then answer: after hospital discharge from surgery, who can help you and how?”).

**Figure 2:**
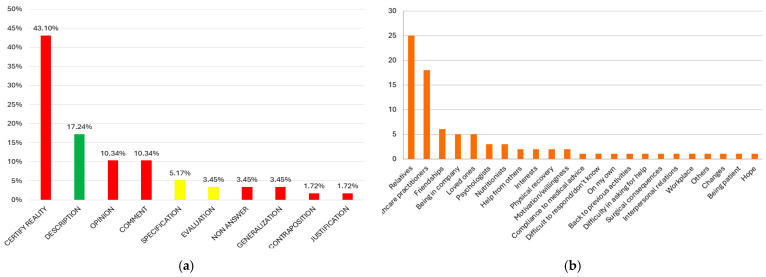
Question 2—After hospital discharge from surgery, who can help you and how? (**a**) Discursive repertories; (**b**) contents.

Similarly to what was observed for the first question, here both upper and lower gastrointestinal patients largely used stabilization repertories, albeit less than in the previous one (as can be noted from Figure 2a).

In particular, the most frequently used repertory was Certify Reality, which covered almost half (43.10%) of the total discursive modalities used in the answers to Question 2. In addition, within the discursive configuration about patients’ resources, there was a greater use of generative repertories (Description in particular) and lesser use of hybrid repertories. Such features of the configuration, representing a scenario more prone to being shared, were noted also from the dW and dM measurements (2.6 and 4.9 respectively).

The resources prospected by patients were family, spouses, partners and healthcare professionals with greater frequency (Figure 2b). Nonetheless, motivation, hope, the clinical condition and physical recovery, and the consequences were semantic dimensions included by patients in characterizing their explanation of the ways by which resources can help in the postoperative period. In particular, these results clinically show how patients entrust the care of specific roles, both formal and informal, and entrust such relationships to be a trigger for managing their recovery.

### 3.3. Describing the Patient’s Participation in Achieving Clinical Objective After Hospital Discharge

The third question (“Once discharged from hospital, how would you manage the postoperative period?”) served to describe the patient’s participation in achieving the clinical objective after hospital discharge.

**Figure 3:**
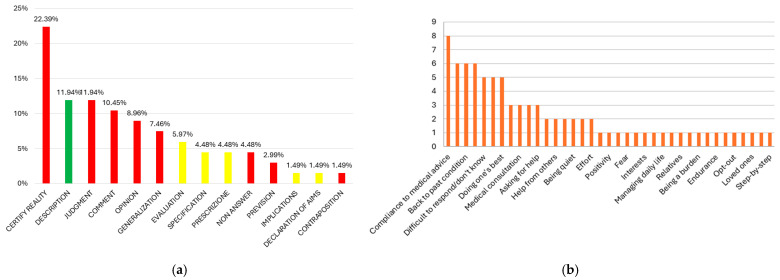
Question 3—Once discharged from hospital, how would you manage the postoperative period? (**a**) Discursive repertories; (**b**) contents.

The argumentative structure of the overall configuration regarding the assessment of postoperative management (dW 2.6 and dM 4.9) resembled the use of resource configuration (Question 2). Indeed, the discursive modalities predominantly used by participants remained stable (Figure 3a): Certify Reality was the most frequently used DR (albeit with a percentage almost halved from the previous question, 22.39%). This discursive modality was used to set postoperative management as an absolute reality and was followed by the generative DR of Description, with a frequency equivalent to that of the stabilization DR Judgment (11.94%) and similar to that of Comment (10.45%). In general, hybrid repertories in the overall configuration of postoperative management accounted for about 20% of the total discursive modalities used: such percentages were higher than those of generative DRs (Figure 3).

In particular, the patients’ configuration structured through these discursive modalities relied on topics such as “compliance with medical advice”, “medical consultations” and “help from others” in general, while the return relied on “to past condition” and “doing one’s best” (Figure 3b). This answer’s results in clinical terms show that the modalities used by patients are partially proper for achieving their clinical objective. At the same time, some prototypical expressions are likely to set a postoperative period characterized by some beliefs in which patients are likely to act stereotypically, with the risk of neglecting some relevant and peculiar aspects in navigating the postoperative period.

### 3.4. Outlining How Patient Adheres to Surgeons’ Prescription

The fourth question was aimed at outlining how the patient would continue to respect the surgeon’s indications: “Please imagine that it has been 3 months since your surgery, in a situation where you have to choose whether or not to do a certain thing. However, it is not certain that this is in line with the indications given to you by the medical staff. What would you take into consideration when making your decision?”.

**Figure 4:**
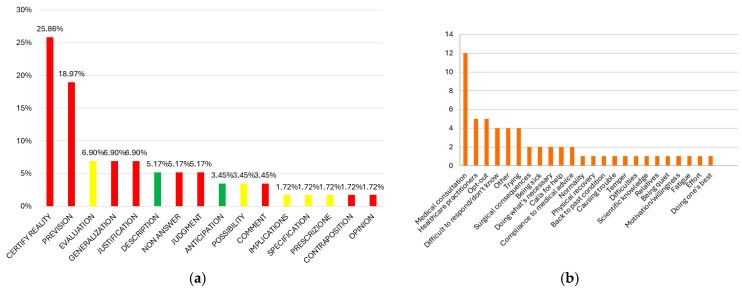
Question 4—Please imagine that it has been 3 months since your surgery, in a situation where you have to choose whether or not to do a certain thing. However, it is not certain that this is in line with the indications given to you by the medical staff. What would you consider when making your decision? (**a**) Discursive repertories; (**b**) contents.

As a response to this question, specifically exploring how patients intend to carry out and pursue the results of cancer surgery, once again patients predominantly used stabilization DRs, with a total frequency that greatly exceeds half of the total discursive modalities used in the fourth question (as shown in Figure 4a). Such DRs account for the values of dW (1.9) and dM (4) for this peculiar configuration.

To be more precise, the first two DRs in order of frequency were Certify Reality and Prevision. Hybrid repertories used the discursive configuration approximately in 15% of discursive processes in the answers, while generative repertories were less used. Interestingly, and unlike the answers to the previous three questions, the answers to the fourth question concerning postsurgical choices featured two different generative discursive modalities configuring various possible situations (Description and Anticipation), not just one. Nevertheless, their total frequency was lower than stabilization and hybrid DRs (Figure 4a).

Focusing on contents, surgical patients highlighted “medical consultations” (that is, contents related to visits, surgical consultations and all those situations in which patients consult their physician) as the discriminant to be used, in accordance with “health practitioners” (physicians, surgeons, nurses in patients’ answers) considered in answers to this question. Also, opting-out and attempts were considered in the decision-making process of patients. Interestingly, “surgical consequences”, “causing trouble”, “physical recovery”, “scientific knowledge”, “effort” (i.e., patients’ initiative and resourcefulness in postoperative adjustment) and “compliance with medical advice” (that is, following instructions given by healthcare professionals) were used less frequently (Figure 4b). Focusing on the decision-making-process, the analysis of the language used by patients outlines themes adequate to the context, missing other possible contents related to trials and error-checking or to a recovery plan. This aspect is corroborated by the general low use of modalities in foreshadowing the critical situation and the related potential choices, although Question 4 makes it possible.

Difficulty in answering and “don’t know” were established by patients in answering Question 4, but also across the other three questions examining the postoperative period.

Furthermore, in all four questions, the observation of the results describing patients’ discursive modalities in relation to descriptive variables (diagnosis, type of intervention) did not permit peculiar language modalities differentiated according to patients’ characteristics to be highlighted.

## 4. Discussion

The research question in this study was based on considering perceived health as a process entailing the possibility of narrating it through natural language. This research question aimed at outlining how upper and lower gastrointestinal surgery is considered by patients when they set their health.

In general, on the basis of this study’s results, the surgical patients’ health configuration was found not to change according to tumor location and consequently to type of surgery.

In particular, expectations for the postoperative period in the patient’s daily life (Question 1) are set as stationary and with a low propensity to change. The chance of modification is solely conveyed by the use of the hybrid DRs of Possibility and Implications. Specifically, through the prevalent use of the Opinion and Prevision DRs together with the Comment DR (i.e., *“what can I know in three months … let’s hope it goes well!”*; *“I’ll see how I will feel, if I’m okay… I hope I’m okay”*), all stabilization modalities, oncological surgery patients established that postoperative scenarios would be the straightforward outcome of the current surgical condition, without considering any interposing event or conduct. Additionally, patients based these expectations on their own opinions or assumptions and on purely personal criteria. Alongside “being sick”, “uncertainty” and “fear”, contents like “being ok” and “hope” or “positivity”, even when considered desirable, further corroborate these discursive modalities, which indicate what will happen in relation to an external event and decrease the patient’s engagement in this postoperative scenario (i.e., *“You will see, they will be determined by the histological examination, I will understand after the things that I have done in those three months. One hopes to be better, of course, but this is determined by histology”*). In this way, the risk for surgical patients after hospital discharge is that they would find themselves unprepared to manage alternative outcomes from the ones currently expected, especially when these outcomes are critical or urgent. Indeed, the theme named “difficult to respond/don’t know” confirms the expectation of the postoperative period outlined by patients that should be explored and implemented with them. For this reason, patients’ perspectives on their future are used to inform subsequent strategies to increase their compliance or reduce patients’ regret (JaKa et al., 2024).

Furthermore, regarding the patient’s use of resources after a surgical operation (Question 2), the predominant use of the Certify Reality DR implies complete certainty of the participants as to which figures they can rely on. This certainty, however, is accompanied by an unclear delineation of how to use these resources and evaluate them (Specification and Evaluation DRs), as well as a failure to indicate (anticipate) alternatives if these figures fail to respond as expected by the patients. This structure is partly mitigated by the use of the Description DR, which, on the other hand, allows participants to specify how to use resources and to specify their potential contribution to the patient’s condition. Themes and specific dimensions involved in patients’ answers show a higher number when compared with the ones of Question 1. In this regard, textual data were collected in a group of patients, 62% of whom had undergone chemotherapy or chemoradiotherapy before surgery and consequently before the present study. Such patients had already experienced consequences of a treatment and this previous experience could be responsible for the variety of contents and responses about resources. Previous experiences can serve as assets for improvement, but their value is further enhanced when they are assessed, specified, re-evaluated and then applied to new settings like the postoperative cancer setting. Therefore, in accordance with other studies (Smith et al., 2021), the various contents (“relatives”, “loved ones”, “psychologist”, “nutritionist”) are listed and patients’ experience can help to identify different roles. Differently from other national and geographical contexts, issues concerning availability or accessibility of services and roles were not present in patients’ answers (English et al., 2024). Nonetheless, postoperative discursive configuration should be based on an assessment of what happened and why choices could be the same and when they could be changed, in consideration of various physical conditions and side effects of treatments. Otherwise, critical implications and difficulties could result (for instance, “on my own” is a specific dimension that examines the risk of not being able to handle the postoperative period, unlike other situations or events).

Furthermore, when examining cancer surgery patients’ discursive configuration in relation to their participation in achieving the clinical objective after hospital discharge (Question 3), patients consider their management mainly by using the Certify Reality DR. Following surgery, cancer patients clearly and certainly establish which elements they are going to use and/or which scenarios will arise. These modalities (expressed for instance in sentences like *“what I will do, work, campaign …”* or *“I had the will to do it, on my own”*) jeopardize management when unexpected events or conditions arise: if the postoperative course changes, these patients will no longer be able to apply these criteria and, therefore, be able to manage the new situation they are facing. In addition, patients use the Judgment DR, through which they characterize what they say with attributes of “correctness” or “that’s how it’s done” in order to support the maintenance of this configuration. In this sense, going “back to past condition” increases such inflexibility, preventing the surgical patient from finding new coping strategies. Similarly, “doing one’s best” is a general content that does not explore effective actions that may be possible after surgery. On the other hand, when using the generative modality Description and considering medical and healthcare professional’s advice through it, participants are able to offer shareable elements (both for themselves and for others) about their situation, which can be shifted to conditions that can be adjusted and used in a versatile way even when future scenarios change. Considering such aspects is mandatory if improvement of surgical decision for high-risk interventions is aimed (Jones et al., 2024).

Lastly, when outlining how patients continue to respect the surgeon’s indications (Question 4), participants outline their future actions mainly through Certify Reality (*“I do so, I trust”*) and Prevision (*“I wouldn’t do it”*) DRs, both of which are stabilization DRs ensuring a highly stable configuration. In other words, they try to see their future actions and behaviors as certain, unambiguous and an inevitable outcome of the current situation. However, the fourth question in the interview focused on a scenario of choices: in answering this question, patients had to refer to their “decision-making process”. This is a point already highlighted in the literature (Fitzgerald et al., 2025; Kinoshita et al., 2023), since shared decision-making and patient trust in their surgeon are key factors: in the present study, the discursive modalities used imply a limited consideration of alternative criteria in decision-making and solutions for imagining themselves in situations different from the one they are currently experiencing or expect to face.

In this narrative structure, a proper and adequate adjustment is given by the hybrid repertory Evaluation, through which participants explain the criteria underlying their choices/decisions (i.e., *“If you are not sure if it is compliant, the first thing to do is to ask the medical team”*). However, even when expressed, these criteria remain on a personal level, so it is uncertain whether they can be understood and applied to other support roles, such as surgeons and the healthcare professionals who initially provided the recommendations.

Facing the features of the scenario presented in the question, “medical consultation” was the predominant dimension considered by patients but interestingly, “surgical consequences”, “physical recovery”, “scientific knowledge” and “effort” were less frequent. Similarly, “compliance with medical advice”, which differs from asking for a consultation since compliance implies the use of a doctor’s knowledge, assessment and indications, was one of the contents less frequently considered. Likewise, “causing trouble”, which is the cause–effect relationship to be considered after highly impacting surgery, was not overriding within patients’ decision-making process. Analyzing the postoperative period eliminates the risk of postoperative decisional regret and improves alignment between surgery and the surgical patient’s role underlying a shared decision-making process (Fitzgerald et al., 2025; Wancata et al., 2016).

Despite following the guidelines for qualitative studies (O’Brien et al., 2014), this study presents some limitations. Apart from the small sample size and the related number of text occurrences (to be increased), the two groups of patients distinguished on the basis of tumor location were not equivalent (upper versus lower gastrointestinal neoplasm). Moreover, a further appropriate distinction should indicate how cancer surgery is considered by patients when they set their health, differentiating between patients who received neoadjuvant chemotherapy or chemoradiotherapy before surgery versus treatment-naïve patients.

In addition to this, “difficulty in answering” and “don’t know” as answers may reflect uncertainty or difficulties in decision-making and would require further in-depth analysis. So, longitudinal studies should analyze the possibility of answering “don’t know” and further investigate it. Despite these limitations, some observations should be made in the surgical context. Primarily, in the early postoperative period, upper and lower gastrointestinal patients base their view of the future postoperative period after hospital discharge on previous experiences, to predict how the future should be and what is expected to support it. Also, patients set surgical convalescence through their own judgements or preconceived notions on surgery or patients requiring cancer surgery, leading to a conditioned perspective on health. However, patients’ participation in their care pathway may vary when various aspects are considered and described through the use of generative modalities. Despite this, the lack of modalities representing the postoperative period by virtue of the healthcare professional’s intentions, the failure to use shared criteria for the analysis of that situation, the lack of representation of probable situations that potentially could occur and the anticipation of surgical implications to be managed, threatens patient’s participation in pursuing surgery. Some studies indicate patients’ participation as the process permitting the management of a clinical event (diagnosis, adverse effects in treatments, acute complications, symptom burden) (Würtz et al., 2023; Collée et al., 2020) as a factor influencing adherence to treatment and the way in which patients can pursue the aim shared with their doctors.

Other studies conducted on gastrointestinal surgery for cancer show that such participation consists chiefly of dialog with the surgeon. In this dialog, treatment can be discussed and the amount of information and content acquired by the patient in interacting with the surgeon can improve the patient’s care pathway (Hermus et al., 2023). Indeed, this information during the dialog can be specified and applied: in this sense, a first meeting with the surgeon is mandatory to describe the surgical operation and the various potential intraoperative choices, and a further meeting between the patient and the healthcare professional is advisable to analyze the consequences on postoperative life and to deal with them beforehand (Würtz et al., 2023).

Similarly, the answers to the question on how patients continue to respect the surgeon’s indications show that oncological surgery patients tend to endure suffering rather than face it and see it as an opportunity to improve their health competences. Moreover, actively engaging caregivers can improve patients’ participation (Pinto et al., 2022).

In light of this, future research should be directed towards the collection of textual data, taking into account both patients and their doctors and caregivers in order to study the features of preoperative communication and to outline preoperative discursive configurations, analyzing them by comparison with postoperative interactive arrangements. Furthermore, as mentioned previously, data should be gathered on the basis of different cancer diagnoses and different types of surgery that patients undergo (upper versus lower gastrointestinal surgery). Research should therefore focus on studies collecting data directly from patient–healthcare interactions, also serving as data to be used with the same patient from the preoperative stage to the subsequent time points (Turchi et al., 2022a).

## 5. Conclusions

Surgery remains the cornerstone of treatment for patients with both upper and lower gastrointestinal cancers, despite the inherent risks of intraoperative and postoperative complications. While surgical outcomes are well-documented, the experience of patients undergoing resection with curative intent has been only partially explored in the literature. To our knowledge, few studies have examined the “narrative configurations” of these patients; even less attention has been specifically directed toward the unique psychological and lived experiences of those undergoing esophageal and major gastrointestinal surgery (Saunders et al., 2021; Reinwalds et al., 2017).

In curative-intent surgery for cancer treatment, in view of the informed consent given by the patient, consideration of patients about surgery has not yet been explored; so, how do gastrointestinal patients pursue the surgical treatment after giving their consent to surgery? Consistently, this study was specifically aimed at outlining how upper and lower gastrointestinal surgery is considered by patients when they set their health. Through a peculiar methodology, in this study we analyzed patients’ categories of knowledge regarding care pathway and discussed the result applied to clinical implications about adjustment to neoplasm and its treatments. According to this study’s findings, surgical patients’ health becomes overwhelmed by a supposed return to a previous condition or the beginning of a disease-free situation. The low use—measured in this study and presented in the previous paragraphs—of discursive modalities suitable for managing the new condition after hospital discharge and the low use of related surgical information, permit some considerations about clinical implications. For instance, surgery is a key point for curative treatments, but when some issues arise in the postoperative period or treatment outcomes, modalities aimed at establishing and not aimed at adjusting to the new conditions can challenge the surgical results. Hence, the unmet need of discerning possible conditions after surgery has been unveiled: in order to improve health in patients’ configuration, they should be aided in anticipating postoperative scenarios, in an attempt to define these scenarios, in establishing challenges in the various alternatives and in considering interposing unexpected events and different behavioral strategies to be adopted.

## Figures and Tables

**Table 1:** Questions and the specific aims they rely upon.

Question Objective	Question
Representing the postoperative period in the patient’s daily life	Please imagine you are at the surgical outpatient clinic about 3 months after surgery: when the surgeon asks you to describe your physical condition with respect to surgery, what might your answer be?
Describing the patient’s use of resources	After hospital discharge from surgery, who can help you and how?
Describing the patient’s participation in achieving clinical objective after hospital discharge	Once discharged from hospital, how would you manage the postoperative period?
Outlining how patient keeps surgeons’ prescription up	Please imagine that it has been 3 months since your surgery, in a situation where you have to choose whether or not to do a certain thing. However, it is not certain that this is in line with the indications given to you by the medical staff. What would you take into consideration when making your decision?

## Data Availability

The original contributions presented in this study are included in the article. Further inquiries can be directed to the corresponding author.

## Abbreviations

The following abbreviations are used in this manuscript:

M.A.D.I.T.	Methodology for the Analysis of Computerized Text Data (in Italian, Metodologia per l’Analisi dei Dati Informatizzati Testuali)

## References

[B1-behavsci-16-00842] Benedict C., Rodriguez V. M., Carter J., Temple L., Nelson C., DuHamel K. (2016). Investigation of body image as a mediator of the effects of bowel and GI symptoms on psychological distress in female survivors of rectal and anal cancer. Support Care Cancer.

[B2-behavsci-16-00842] Byrne B. E., Siaw-Acheampong K., Evans O., Taylor J., Huddy F., Nilsson M., Griffiths E. A., Low D., Gossage J., Dunn J., Zeki S., Markar S., Avery K., Blazeby J. M., Cockbain A., Moss C., van Hemelrijck M., Andreyev J., Davies A. R., on behalf of the RESTORE Delphi study group (2024). REsolution of Symptoms afTer Oesophago-gastric cancer REsection delphi (RESTOREd)-standardizing the definition, investigation and management of gastrointestinal symptoms and conditions after surgery. British Journal of Surgery.

[B3-behavsci-16-00842] Collée G. E., van der Wilk B. J., van Lanschot J. J. B., Busschbach J. J., Timmermans L., Lagarde S. M., Kranenburg L. W. (2020). Interventions that facilitate shared decision-making in cancers with active surveillance as treatment option: A systematic review of literature. Current Oncology Reports.

[B4-behavsci-16-00842] English N. C., Ivankova N. V., Smith B. P., Jones B. A., Herbey I. I., Rosamond B., Kim D. H., Oslock W. M., Schoenberger-Godwin Y. M., Pisu M., Chu D. I. (2024). Providers’ and survivors’ perspectives on the availability and accessibility of surgery in gastrointestinal cancer care. Journal of Gastrointestinal Surgery.

[B5-behavsci-16-00842] Färnqvist K., Mälberg K., Johar A., Schandl A., Lagergren P. (2024). Trajectories of patient-reported outcomes after oesophageal cancer surgery—A population-based study. European Journal of Cancer.

[B6-behavsci-16-00842] Fitzgerald J. E., Austin P., Monton O., Bechthold A. C., Kopecky K. E. (2025). Patient regret after gastrointestinal surgery for cancer: A narrative review. Journal of Surgical Oncology.

[B7-behavsci-16-00842] Gao L., Chen W., Qin S., Yang X. (2024). The impact of preoperative interview and prospective nursing on perioperative psychological stress and postoperative complications in patients undergoing TACE intervention for hepatocellular carcinoma. Medicine.

[B8-behavsci-16-00842] Hermus M., van der Wilk B. J., Chang R., Dekker J. W. T., Coene P. L. O., Nieuwenhuijzen G. A. P., Rosman C., Heisterkamp J., Hartgrink H. H., Timmermans L., Wijnhoven B. P. L., van der Zijden C. J., van Lanschot J. J. B., Busschbach J., Lagarde S. M., Kranenburg L. W. (2023). Esophageal cancer patients’ need for information and support in making a treatment decision between standard surgery and active surveillance. Cancer Medicine.

[B9-behavsci-16-00842] Im D., Pyo J., Lee H., Jung H., Ock M. (2023). Qualitative research in healthcare: Data analysis. Journal of Preventive Medicine and Public Health.

[B10-behavsci-16-00842] JaKa M. M., Henderson M. G., Alch S., Ziegenfuss J. Y., Zinkel A. R., Osgood N. D., Werner A., Borgert Spaniol C. M., Flory M., Mabry P. L. (2024). Qualitative interviews to add patient perspectives in colorectal cancer screening: Improvements in a learning health system. Journal of Cancer Education.

[B11-behavsci-16-00842] Jones T. S., Mckown L., Lane A., Horney C., Unruh M., Brown N., Sommerville-Henderson S., Jones E. L., Albright K., Levy C., Robinson T. (2024). Patient participation in multidisciplinary high-risk surgery discussions: A pilot study. Journal of Palliative Medicine.

[B12-behavsci-16-00842] Khalil M., Woldesenbet S., Altaf A., Rashid Z., Zindani S., Thammachack R., Husain S., Pawlik T. M. (2025). Employment disruption and financial burden associated with gastrointestinal cancers. Annals of Surgical Oncology.

[B13-behavsci-16-00842] Kinoshita H., Nishigori T., Nakabe T., Shimoike N., Sato K., Imanaka Y., Obama K., Matsumura Y. (2023). Factors associated with postoperative decisional regret in patients undergoing gastrointestinal cancer surgery: A prospective cohort study. The American Surgeon.

[B14-behavsci-16-00842] Konradsson M., van Berge Henegouwen M. I., Bruns C., Chaudry M. A., Cheong E., Cuesta M. A., Darling G. E., Gisbertz S. S., Griffin S. M., Gutschow C. A., van Hillegersberg R., Hofstetter W., Hölscher A. H., Kitagawa Y., van Lanschot J. J. B., Lindblad M., Ferri L. E., Low D. E., Luyer M. D. P., Nilsson M. (2020). Diagnostic criteria and symptom grading for delayed gastric conduit emptying after esophagectomy for cancer: International expert consensus based on a modified Delphi process. Diseases of the Esophagus.

[B15-behavsci-16-00842] Markar S. R., Zaninotto G., Castoro C., Johar A., Lagergren P., Elliott J. A., Gisbertz S. S., Mariette C., Alfieri R., Huddy J., Sounderajah V., Pinto E., Scarpa M., Klevebro F., Sunde B., Murphy C. F., Greene C., Ravi N., Piessen G., Hanna G. B. (2022). Lasting Symptoms After Esophageal Resection (LASER): European multicenter cross-sectional study. Annals of Surgery.

[B16-behavsci-16-00842] McDermott F. D., Heeney A., Kelly M. E., Steele R. J., Carlson G. L., Winter D. C. (2015). Systematic review of preoperative, intraoperative and postoperative risk factors for colorectal anastomotic leaks. British Journal of Surgery.

[B17-behavsci-16-00842] Morgan E., Arnold M., Gini A., Lorenzoni V., Cabasag C. J., Laversanne M., Vignat J., Ferlay J., Murphy N., Bray F. (2023). Global burden of colorectal cancer in 2020 and 2040: Incidence and mortality estimates from GLOBOCAN. Gut.

[B18-behavsci-16-00842] O’Brien B. C., Harris I. B., Beckman T. J., Reed D. A., Cook D. A. (2014). Standards for reporting qualitative research: A synthesis of recommendations. Academic Medicine.

[B19-behavsci-16-00842] Pinto E., Alfieri R., Orrù L., Dalla Riva M. S., Turchi G. P. (2022). Forward to a methodological proposal to support cancer patients: The dialogics’ contribution for the precision care. Medical Oncology.

[B20-behavsci-16-00842] Reinwalds M., Blixter A., Carlsson E. (2017). A Descriptive, qualitative study to assess patient experiences following stoma reversal after rectal cancer surgery. Ostomy Wound Management.

[B21-behavsci-16-00842] Rupp S. K., Stengel A. (2021). Influencing factors and effects of treatment on quality of life in patients with gastric cancer—A systematic review. Front Psychiatry.

[B22-behavsci-16-00842] Saunders C. H., Goldwag J. L., Read J. T., Durand M. A., Elwyn G., Ivatury S. J. (2021). ‘Because Everybody is so Different’: A qualitative analysis of the lived experiences and information needs of rectal cancer survivors. BMJ Open.

[B23-behavsci-16-00842] Smith D. T., Barrett J., Acher A. W., Joachim A., Huynh B., Schreiter N., Stafford L. C., Abbott D. E., Alagoz E. (2021). Patient preferences for GI cancer surveillance and telemedical follow-up. Surgical Oncology.

[B25-behavsci-16-00842] Turchi G. P., Dalla Riva M. S., Ciloni C., Moro C., Orrù L. (2021). The interactive management of the SARS-CoV-2 virus: The social cohesion index, a methodological-operational proposal. Frontiers in Psychology.

[B26-behavsci-16-00842] Turchi G. P., Fabbian A., Alfieri R., Da Roit A., Marano S., Mattara G., Pilati P., Castoro C., Bassi D., Dalla Riva M. S., Orrù L., Pinto E. (2022a). Managing the consequences of oncological major surgery: A short- and medium-term skills assessment proposal for patient and caregiver through M.A.D.I.T. methodology. Behavioral Sciences.

[B27-behavsci-16-00842] Turchi G. P., Orrù L., Iudici A., Pinto E. (2022b). A contribution towards health. Journal of Evaluation in Clinical Practice.

[B24-behavsci-16-00842] Turchi G. P., Romanelli M., Bonazza F., Girardi A. (2014). Discursive configuration. Encyclopedia of critical psychology.

[B28-behavsci-16-00842] Turchi G. P., Salvalaggio I., Croce C., Dalla Riva M. S., Orrù L., Iudici A. (2022c). The health of healthcare professionals in Italian oncology: An analysis of narrations through the MADIT methodology. Behavioral Sciences.

[B29-behavsci-16-00842] Wancata L. M., Hinshaw D. B. (2016). Rethinking autonomy: Decision making between patient and surgeon in advanced illnesses. Annals of Translational Medicine.

[B30-behavsci-16-00842] Würtz H. J., Rahr H. B., Lindebjerg J., Edwards A., Steffensen K. D. (2023). Impact of an in-consult patient decision aid on treatment choices and outcomes of management for patients with an endoscopically resected malignant colorectal polyp: A study protocol for a non-randomised clinical phase II study. BMJ Open.

[B31-behavsci-16-00842] Xi Y., Xu P. (2021). Global colorectal cancer burden in 2020 and projections to 2040. Translational Oncology.

